# Supplementary figures and images for: Comparison of Machine Learning Models for Brain Age Prediction Using Six Imaging Modalities on Middle-Aged and Older Adults
Source: Sensors (Basel). 2023 Mar 30;23(7):3622. doi: 10.3390/s23073622 (PMC10098634; doi:10.3390/s23073622)

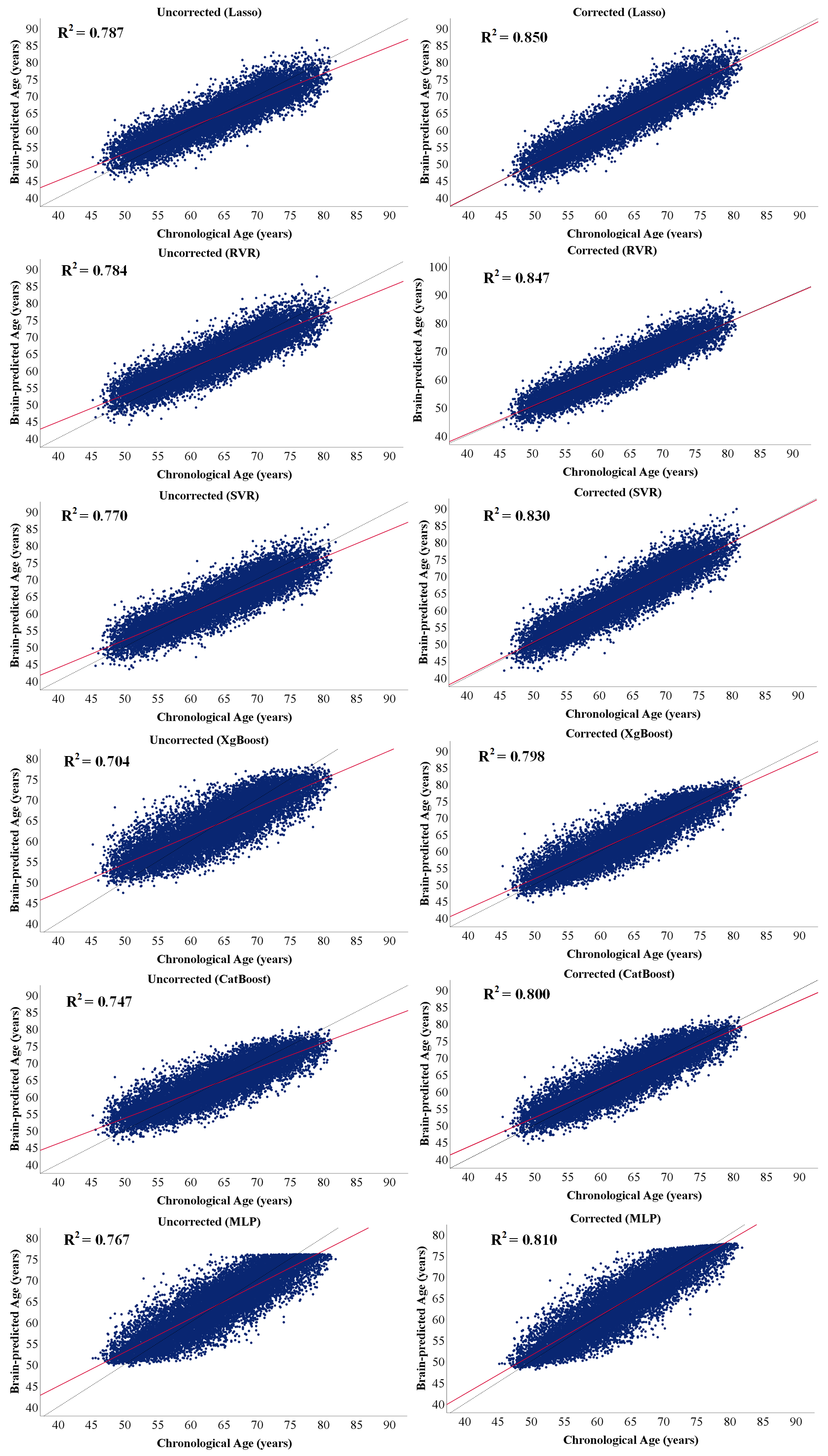

Supplement: Supplementary file 1 [file sensors-23-03622-s001.zip › sensors-2302474-supplementary.tif]
